# Prevention of sickness absence through early identification and rehabilitation of at-risk patients with musculoskeletal disorders (PREVSAM): 12-month follow-up of a randomised controlled trial

**DOI:** 10.1186/s12891-026-09859-x

**Published:** 2026-04-27

**Authors:** Maria EH Larsson, Annika Ekhammar, Rode Grönkvist, Lena Bornhöft, Lena Nordeman, Kristina Holmgren, Anna Grimby-Ekman, Gunnel Hensing, Cecilia Björkelund, Stefan Bergman, Maria Dottori, Susanne Bernhardsson

**Affiliations:** 1 Research, Education, Development & Innovation, Primary Health Care, Region Västra Götaland, Gothenburg, Sweden; 2 Department of Health and Rehabilitation/Unit of Physiotherapy, Institute of Neuroscience and Physiology, The Sahlgrenska Academy, University of Gothenburg, Gothenburg, Sweden; 3 Närhälsan Eriksberg Primary Care Rehabilitation center, Region Västra Götaland, Göteborg, Sweden; 4 School of Public Health and Community Medicine, Institute of Medicine, The Sahlgrenska Academy at University of Gothenburg, Gothenburg, Sweden; 5 General Practice/Family Medicine, School of Public Health and Community Medicine, Institute of Medicine, Sahlgrenska Academy, University of Gothenburg, Gothenburg, Sweden; 6 Department of Health and Rehabilitation/Unit of Occupational Therapy, Institute of Neuroscience and Physiology, The Sahlgrenska Academy, University of Gothenburg, Gothenburg, Sweden; 7 Spenshult Research and Development Centre, Halmstad, Sweden

**Keywords:** Musculoskeletal pain, Physiotherapy, Occupational therapy, Interdisciplinary teamwork, Primary care, Secondary prevention, Sickness absence

## Abstract

**Background:**

The team-based, interdisciplinary rehabilitation model, PREVention of Sickness Absence for Musculoskeletal disorders (PREVSAM), was developed and evaluated in a Swedish primary care context. The purpose of this follow-up study was to assess 12-month effects of rehabilitation according to the PREVSAM model on sickness absence and patient-reported health outcomes in patients initially assessed as being at increased risk for sickness absence.

**Methods:**

This was a two-armed randomised controlled trial at eight primary care rehabilitation clinics, comparing rehabilitation according to the PREVSAM model with treatment as usual (TAU). In this 12-month follow-up, sickness absence and patient-reported health outcomes were assessed in 249 participants. Analyses were performed using logistic regression, zero-inflated negative binomial regression, and Cox regression.

**Results:**

Most participants remained in full- or part-time work (PREVSAM 67.7%, TAU 60.7%) and had zero registered sickness benefit days during the 12 months’ follow-up period (PREVSAM Md;0; IQR 0-10.5, TAU 0;0-20.4), with no statistically significant difference between the groups. Improvements over time were seen in all patient-reported outcomes in both groups, but no significant between-group differences were found.

**Conclusions:**

The PREVSAM model was not shown to be more effective than TAU in reducing sickness absence amongst patients with musculoskeletal disorders who were identified to be at risk for long-term sick leave or to reduce risk for sickness absence. Patient-related health outcomes improved significantly over time in both groups, suggesting that the interventions, regardless of type, contributed positively to participants’ health and recovery. The findings are nevertheless encouraging as most participants—regardless of group—remained in full- or part-time work, with no registered sickness benefit days during the 12 months’ follow-up period. This finding suggests that most individuals who initially present with risk factors for long-term pain and sick leave do not go on to develop prolonged sickness absence.

**Trial registration:**

The trial was registered with ClinicalTrials.gov, Protocol ID: NCT03913325. Study Registration Date, First Submitted 09/04/2019, First Posted 12/04/2019.

**Supplementary Information:**

The online version contains supplementary material available at 10.1186/s12891-026-09859-x.

## Introduction

Musculoskeletal disorders (MSDs) are well known to impact on peoples’ lives as they can significantly affect activities of daily living and work ability. Osteoarthritis and low back pain are the two most prevalent categories of MSDs, with prevalence in the Nordic countries estimated at 11–14% and almost 6%, respectively [1]. Primary care serves as the initial level of care for MSDs [2], which, together with mental disorders, are the most common reasons for sickness absence in Sweden. Musculoskeletal disorders accounted for 16% and 21% for women and men, respectively. The corresponding figures for mental disorders were 54% of all cases among women and 41% among men. Mental disorders are the most common diagnosis in all age groups except for men over 60 years of age, for whom musculoskeletal disorders are the most common [3].

A bi-directional correlation between musculoskeletal pain and mental disorders, such as depression and anxiety, is often present regardless of whether the pain is acute/subacute or chronic [4]. Level of disability, unemployment, widespread pain, a high level of pain, and catastrophising are prognostic indicators associated with long-term disability in both acute/subacute and chronic low back pain subgroups in primary care [5]. The challenges in providing effective treatments for complex MSDs are well known [6, 7], and the recognition that biopsychosocial risk factors influence the recovery process in MSDs has led to the development of various approaches aimed at optimising treatment [8]. In a stepped care model, more comprehensive treatments are only offered to people if less extensive interventions lead to inadequate responses [9]. Risk-stratified care aims to identify patients with higher predicted risk of poor outcome and directly offer specific treatments. Identifying and assessing patients’ risk of developing long-term problems is therefore needed [10, 11]. Those at lower risk can be provided fewer or less comprehensive interventions and be reassured that they have a good prognosis, while those at higher risk can be offered more comprehensive, team-based interventions aiming to prevent long-term problems.

In MSD care, assessments and treatments provided by a physiotherapist or a general practitioner are first line care, and many patients will recover satisfactorily. To optimise care for those who have developed long-term problems affecting quality of life and work ability, multi-professional rehabilitation models may be useful [12, 13]. The overall aim of this type of rehabilitation is to provide strategies for being able to live a good life with and despite pain, rather than solely focusing on eliminating the pain. The rehabilitation should be provided with a person-centred approach and a biopsychosocial view on the complexity of living with pain. Within this biopsychosocial framework, work participation is considered a central domain of functioning, alongside physical and psychological health. Work ability is generally viewed as reflecting the fit between an individual’s physical, mental, and psychosocial resources and the demands of the job [14]. Interdisciplinary team-based rehabilitation has been proven beneficial for patients with complex pain problems, as it can enhance both physical and psychological functioning, as well as work ability [15, 16]. It can also promote shared responsibility amongst the team members and foster a deeper understanding of the patients’ conditions.

There is a knowledge gap regarding whether early identification followed by early interdisciplinary teamwork in primary care can prevent long-term problems and sickness absence in patients with MSDs. To fill this gap, we developed the rehabilitation model ‘Prevention of sickness absence through early identification and rehabilitation of at-risk patients with musculoskeletal disorders’ (PREVSAM) [17]. The PREVSAM model addresses key prognostic factors for sickness absence based on previous literature and theories, by combining early identification of high-risk patients with structured, team-based rehabilitation. Psychological risk factors are managed through optional early access to psychological treatment, while work-related barriers are targeted through the possibility of workplace contact and support for adjustments. A coordinated interdisciplinary team creates a person-centred rehabilitation plan with clearly defined responsibilities, ensuring consistent guidance and reducing fragmentation in care. This structured approach also strengthens patient engagement and self-management strategies. Together, these components aim to reduce the risk of prolonged sickness absence by addressing clinical, psychological, and work-environment factors using an integrated and individualised approach.

We conducted a randomised controlled trial (RCT), where rehabilitation according to the PREVSAM model was compared to treatment as usual (TAU). In our short-term follow-up (3 months), the PREVSAM intervention was not proven statistically superior to TAU using a significance level of *p* < 0.05 [17]. However, the proportion of participants remaining in full- or part-time work was higher in the PREVSAM group. The overall aim of the present study was to evaluate long-term (12 months) effects of the PREVSAM intervention on sickness absence and patient-reported health outcomes compared with TAU.

Research questions answered in this manuscript:

- RQ1: Over a 12-month follow-up period, is rehabilitation according to the PREVSAM model more effective than TAU in preventing sickness absence in patients with musculoskeletal pain identified as being at increased risk for sickness absence?
- RQ2: Over a 12-month follow-up period, does the self-reported risk for sickness absence decrease after rehabilitation according to the PREVSAM model, compared with TAU?
- RQ3. Over a 12-month follow-up period, do patients treated according to the PREVSAM model report higher work ability, improved pain status, better self-efficacy, less physical disability, decreased self-reported level of anxiety and depression symptoms or higher health-related quality of life, than patients who received TAU?

## Materials and methods

### Study design

We conducted a two-armed RCT at eight primary care rehabilitation clinics in Western Sweden and evaluated the effects of rehabilitation according to the PREVSAM model compared with TAU. In this study, we report findings from the 12-month follow-up regarding sickness absence, patient-reported work ability and health outcomes according to the CONSORT Checklist. We have described setting and methods in detail in previous study protocol [17] and short-term (3 months) effects [18] articles, and how the PREVSAM model was implemented, mechanisms of impact, and contextual factors in a process evaluation article [19]. There was limited patient or public involvement in the design, conduct and reporting of the trial.

### Recruitment

The recruitment started in May 2019 and was terminated in June 2022. Patients of working age seeking care for MSD were asked at their first consultation to the rehabilitation clinic to fill out the Örebro Musculoskeletal Pain Screening Questionnaire, Short Form (ÖMPSQ-SF) [20]. Those who scored 40 points or more, indicating an increased risk of sickness absence, were assessed against the predefined inclusion end exclusion criteria (Table 1). It was mandatory to have an eligible source of income for insurance by the Swedish social insurance agency (SSIA). The income could come from a paid job, parental leave benefits, unemployment benefits, or student aid. While fluency in Swedish was not required, participants with insufficient literacy to complete the study questionnaires were excluded.

**Table 1:** Inclusion and exclusion criteria

Inclusion criteria	Exclusion criteria
Musculoskeletal disorders with less than three months duration	“Red flags” indicating risk for serious pathology or pain not primarily from the musculoskeletal system
Adult of working age (aged ≥ 18 years)	Fully retired or receiving full disability pension
≥ 40 points on Örebro Musculoskeletal Pain Screening Questionnaire -Short Form	Sick leave due to the musculoskeletal disorder > 30 days during the last year
Having an income insured by the Swedish sickness insurance system	Severe mental illness and/or ongoing substance abuse disorders
	Pregnancy
	Insufficient Swedish literacy

### Randomisation procedure

Patients who met the inclusion criteria and did not present any exclusion criteria were provided with verbal and written information explaining that the trial compared TAU with a more extensive team-based rehabilitation. They were informed that they would be randomised and aware of their group allocation. If they consented to participate, they were randomised at the rehabilitation clinic or by phone a few days later by a physiotherapist or occupational therapist participating in the trial. We, the researchers, had prepared the randomisation and provided the participating clinics with sealed opaque envelopes containing written allocation to either PREVSAM or TAU. The randomisation was produced by a computer programme using block-randomisation in blocks of six with an allocation ratio of 1:1 (three each) [21].

### The PREVSAM model

The main goal for the intervention, rehabilitation according to the PREVSAM model, was to prevent sickness absence and long-term pain problems. In the PREVSAM model, a person-centred approach is used and the physiotherapist, occupational therapist, and the patient themselves, are all active team members. First, all patients were offered individual consultations with the physiotherapist and the occupational therapist, who make their professional assessments. Subsequently, at a team meeting, the three of them co-create an individual health plan based on these assessments from a biopsychosocial perspective of the patient’s situation. The patient’s narrative and priorities of healthy lifestyle behaviours are discussed, together with the healthcare professionals’ perspectives. The health plan is a living document with the patient’s goal and milestones, describing actions to be taken and planned follow-ups. Moreover, the team discussed role responsibilities and encouraged the patient to contact a psychotherapist to address psychosocial concerns, who would then be invited to join the team, or, when appropriate, to obtain a medical assessment from a general practitioner. The team also engaged other relevant stakeholders when needed and, following an assessment of the patient’s work situation, offered to contact the workplace to formulate concrete work adjustments.

### Treatment as usual

Treatment as usual comprised standard physiotherapy delivered within primary care, based on the physiotherapist’s clinical judgment. Occupational therapy was offered if considered appropriate.

### Data collection

#### Background variables and treatments delivered

Data regarding age, sex, living situation, education, occupation, income, and country of birth were collected at baseline using a web-based questionnaire, accessed via a unique link in an e-mail sent to the participants (esMaker.net, ©Entergate, Halmstad, Sweden). Data regarding diagnosis, number of consultations, duration of rehabilitation period, and treatments delivered were collected from the healthcare professionals’ treatment reports at each rehabilitation clinic after completion of treatment. Treatment reports included the Swedish Classification of Health Interventions procedure codes registered for each patient on each consultation, describing patient-oriented actions for investigative, treatment, or preventive purposes [22]. There is only a limited hierarchy in the classification, and procedure codes are regularly removed, replaced, or added, by the National Board of Health and Welfare. They may also differ amongst Swedish regions, making comparison difficult. Previous research has provided suggestions for categorisation [23], which guided our categorisations [19].

Data on number of psychological treatments were collected from the psychotherapist.

#### Primary outcome

The primary outcome, sickness absence, was operationalised and data were retrieved in accordance with proposed outcome measures from the Swedish Social insurance report 2016:9 [24]:

- Number and proportion of individuals who were on sickness absence and the number and proportion of individuals who remained in full- or part-time work during the follow-up period.
- Number of gross and net sick-leave days during the intervention and follow-up period.
- Time to first sickness absence spell.

These data are based on individual level sick-leave records from the 12 months following inclusion, including diagnoses, for all sick-leave spells longer than 14 days in which sickness cash benefits were provided by the SSIA. Although the study’s inclusion criteria generally imply that sickness absence is related to MSDs, such as “Diseases of the musculoskeletal system and connective tissue” (M) and “Injuries, poisonings, and certain other consequences of external causes” (S), there can also be associated symptoms or comorbidities classified within “Mental and behavioural disorders” (F) or “Symptoms, signs of disease and abnormal clinical and laboratory findings not elsewhere classified” (R). Data were retrieved from the MicroData for Analysis of the Social Insurance System (MiDAS) database.

In addition to data on sickness absence from the SSIA, self-reported responses to weekly text messages via mobile phone on sickness absence (SMS-Track ApS, Denmark, www.sms-track.com) were collected to analyse short-term absence, i.e. sick-leave days not compensated by the SSIA. The participants entered a number from 0 to 7 in response to the question “Have you been absent from work during the past week due to your musculoskeletal pain, and if so for how many days?

#### Secondary outcomes

A range of secondary outcomes was specified, covering several aspects of health and collected using patient-reported outcome measures (PROMs) included in the web-based questionnaire at baseline and at one, three, six, and 12 months after inclusion. The PROMs included risk for sickness absence, work ability, physical disability, musculoskeletal pain intensity and frequency, general and pain self-efficacy, depression and anxiety symptoms, and health-related quality of life. Please see Study protocol [17] for full description of outcome measures.

### Data analysis and statistical methods

#### Primary outcomes

The proportion of individuals who remained in full- or part-time work was analysed using logistic regression. Gross and net days of sickness absence were analyzed using zero-inflated negative binomial regression [25]. This model was chosen as the numbers of days of sickness absence were count variables that exhibited clear overdispersion as well as a strong concentration at zero. Zero-inflated negative binomial regression models have two effect parameters of intervention: one affecting the probability of having zero sick days and the other affecting the predicted mean number of sickness absence days in the non-zero part of the distribution. To interpret the modeled effect of the intervention, both these parameters must be considered. The “pscl” R package was used for zero-inflated negative binomial regression [26]. Time to first sickness absence spell was analysed using Cox regression, as this outcome was concerned with time-to-event data. Self-reported sickness absence was analysed using descriptive statistics and is presented with means and 95% confidence intervals. Due to a considerable amount of missing data, further statistical analysis of self-reported sickness absence (collected via SMS-Track) was not performed. All primary analyses were carried out using the R statistical computation software, version 4.4.1 [27].

#### Secondary outcomes

All secondary outcomes were analysed using generalised linear mixed models. They were all modelled as linear (using an identity link), except for pain frequency, which was considered ordinal with 4 levels and modelled using a cumulative link logit mixed model [28]. The “ordinal” R package was used for the cumulative link logit mixed model [29]. Analyses of secondary outcomes modelled as linear were carried out in SPSS (version 29.0). Analysis of pain frequency was carried out in R (version 4.4.1).

Analysis was by intention to treat.

The trial was registered with ClinicalTrials.gov, Protocol ID: NCT03913325. Study Registration Date, First Submitted 09/04/2019, First Posted 12/04/2019.

## Results

### Participants

**Fig. 1:**
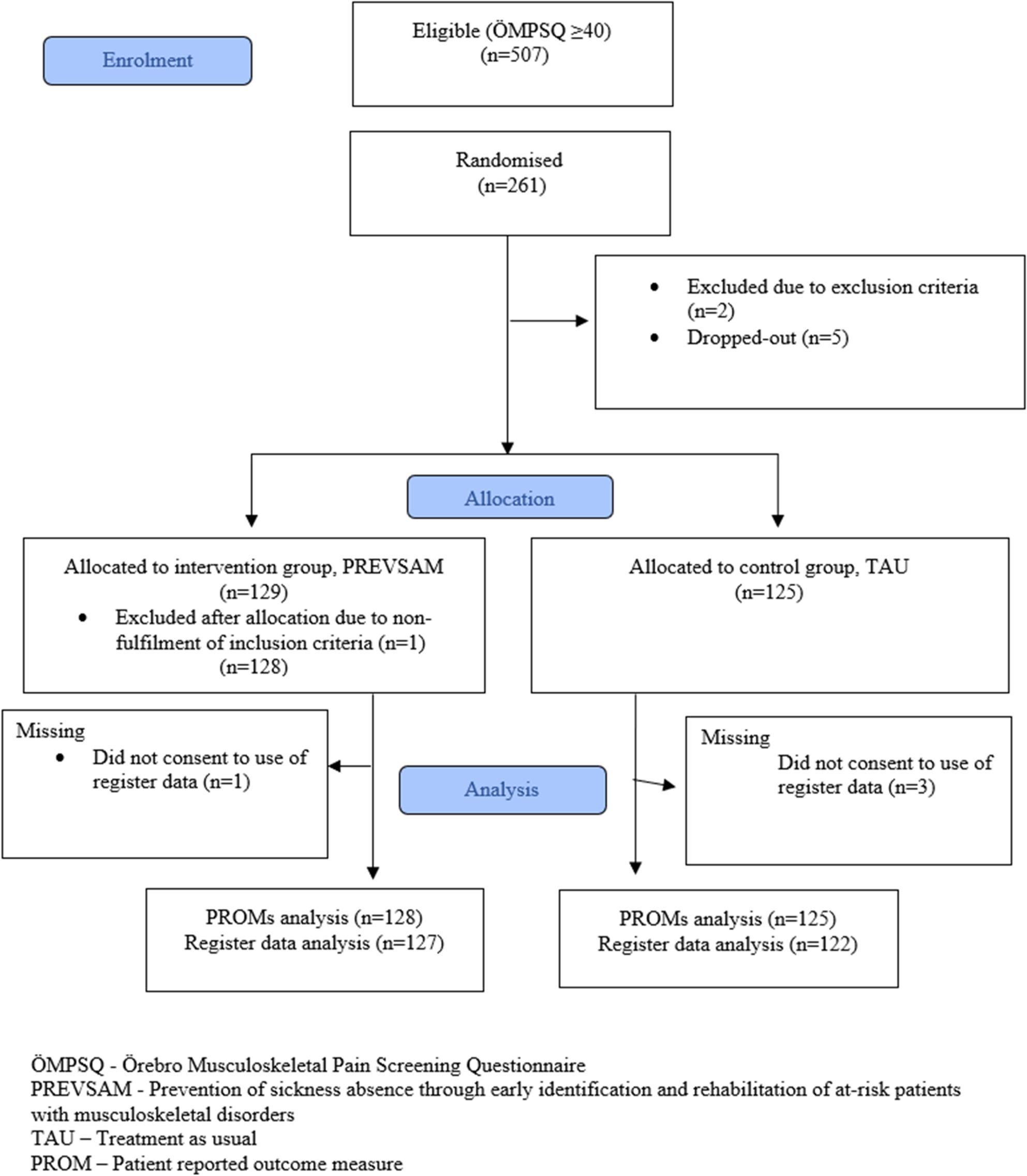
Flow chart of participants' enrolment and allocation

A total of 253 participants were included in this long-term follow-up study: 128 were randomised to rehabilitation according to the PREVSAM model and 125 to TAU. Four participants withdrew their consent for access to register data, leaving 127 participants in the PREVSAM group and 122 in the TAU group for analysis of the primary outcome sickness absence (Fig. 1). Even though the inclusion time was extended, the included number of participants did not fully reach the required sample size of at least 264 (132 individuals in each group), as suggested by the power calculation [17]. The recruitment period was stopped when several participating rehabilitation clinics were no longer able to participate due to staffing difficulties during the COVID-19 pandemic.

### Treatments provided

Number of consultations, follow-ups by phone and treatment weeks are presented in Table 2. Physiotherapy was the most common treatment, provided to more than 95% in both the PREVSAM group and in TAU. As the PREVSAM model is based on team-rehabilitation, the frequency of cases with occupational therapist contact was, as expected, much higher in the PREVSAM group (over 80%) than in TAU (approximately 20%). In the PREVSAM group, the median number of physiotherapy treatment sessions was five, compared with four in TAU. Team meetings were also an essential component in the PREVSAM model, reported in 86% of participants’ treatment reports in the PREVSAM group. Team meetings were uncommon within TAU, occurring in only 3% of cases. A higher proportion received follow-ups by phone in the PREVSAM group. The lengths of the treatment periods varied substantially in both groups but were on average greater in the PREVSAM group.

**Table 2:** Comparison of number of consultations, follow-ups by phone and treatment weeks

	Consultation to physiotherapist	Consultation to occupational therapist	Team-meeting	Follow-up by phone	Number of treatment weeks
Intervention	*n* = 127	*n* = 105	*n* = 110	*n* = 75	
*n* = 128	99%	81%	86%	59%	
Mean (min-max)	7.0 (0–54)	1.9 (0–14)	1.7 (0–4)	2.0 (0–7)	22.0 (1–65)
Median (Q1;Q3)	5 (3;9)	1 (1;2)	2 (1;2)	2 (1;2)	19 (13;28.75)
Treatment as usual	*n* = 120	*n* = 26	*n* = 4	*n* = 41	
*n* = 125	96%	21%	3%	33%	
Mean (min-max)	6.3 (0–43)	3.4 (0–10)	1.5 (0–2)	1.9 (0–7)	12.8 (5–18)
Median (Q1;Q3)	4 (2;7.75)	2 (1;6)	1.5 (1;2)	2 (1;2)	9 (1;71)

Procedure codes, compiled into broader categories, are presented in Table 3. Almost all participants in both groups were assigned procedure codes for assessment. When comparing procedure codes, significantly more assessments were registered in the PREVSAM group; being examined by both a physiotherapist and an occupational therapist contributed to the higher number of assessments. The use of behavioural medicine interventions and work-related advice and interventions were reported to a statistically significantly higher level in the PREVSAM group than in TAU, which is in line with the PREVSAM model’s aim. In addition, patient-related external administration was significantly higher in the PREVSAM group.

**Table 3:** Reported procedure codes^a^ used during treatment period

Treatment category	PREVSAM group *n* = 128		TAU group *n* = 125		Between-group analysis χ^2^
Proportion who received treatment	n	%	n	%	p-value^b^
Assessment	125	97.7	121	96.8	0.678
Number of assessments 0 1 2 3 4 5 6	3 18 51 30 18 6 2	2.3 14.1 39.8 23.4 14.1 4.7 1.6	4 86 21 10 2 2 0	3.2 68.8 16.8 8.0 1.6 1.6 0	**< 0.001**
Physical activity ^1^ Advice and exercise	113	88.3	117	93.6	0.141
Health plan/ rehabilitation plan	117	91.4	20	16	**< 0.001**
Body awareness ^2^	35	27.3	29	23.2	0.448
Behavioural medicine interventions ^3^	37	28.9	7	5.6	**< 0.001**
Information and education ^4^	81	63.3	66	52.8	0.091
Manual treatment	5	3.9	7	5.6	0.526
Physical modalities ^5^	42	32.8	35	28.0	0.406
Medical aids ^6^	24	18.8	31	24.8	0.243
Work-related advice and interventions	80	62.5	14	11.2	**< 0.001**
Patient-related external administration	11	8.5	2	1.6	**0.012**

^a^Multiple treatment codes could be applied within the same consultation

^b^significance level 0.05, significant values in bold

^1^Advice and exercises to increase physical capacity through, for example, muscle function and strength training, cardiovascular training, and mobility training

^2^Training to improve body perception and body image, for example Basic Body Awareness Training, yoga, relaxation training and rhythm-and-movement exercises

^3^Strategies to prevent, treat, or manage health problems, for example stress management, mindfulness, motivational interviewing

^4^Information and education about, for example, health, ill-health, self-care, lifestyle habits and pain

^5^Acupuncture, TENS, laser therapy, ultrasound examination

^6^Crutch, rollator/walker, orthosis, reacher/grab stick

In the PREVSAM model, a workplace dialogue between healthcare, the employee, and the employer aiming to create a shared understanding was offered to identify barriers and formulate concrete work adjustments. However, around 60% reported that their MSD did not affect their work, and almost 15% stated that they did not want to inform their employer about their condition. For others, the opportunity to discuss their rights and the employer’s obligations regarding workplace accommodation was sufficient. Direct employer contact by the PREVSAM team occurred in only about 5% of all cases. An additional 10% chose to initiate the contact themselves after receiving this guidance [19].

### Primary outcome

#### Registered sickness benefit days

A majority of the participants remained in full- or part-time work and had zero registered sickness benefit days during the 12-month follow-up period. The PREVSAM model was not shown to be more effective than TAU in preventing sickness absence, in patients with MSDs who had been identified as being at increased risk for sickness absence.

**Fig. 2:**
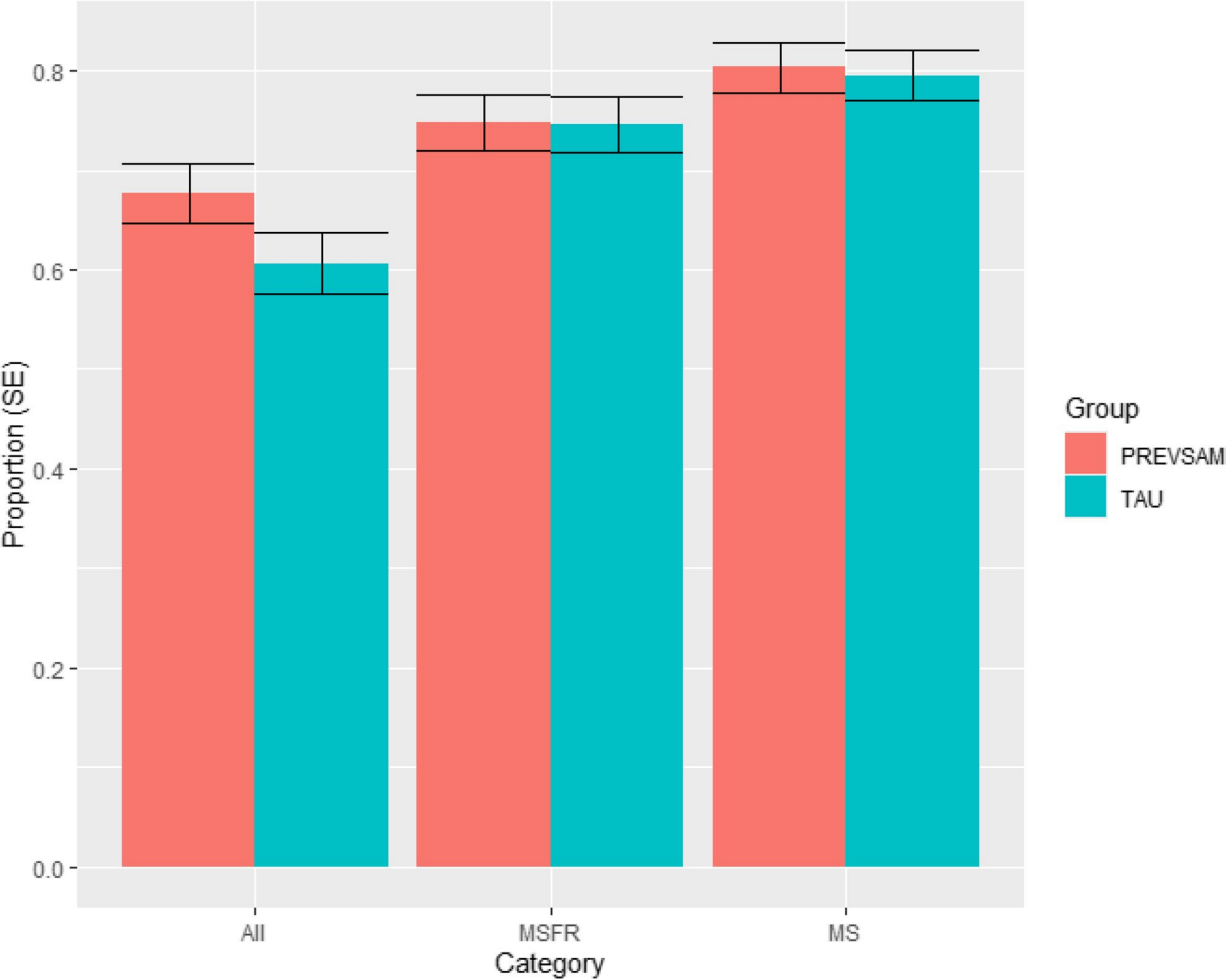
Bar plot of proportion remaining in work at 12 months (no registered sickness benefit days), per sickness absence diagnoses and trial arm. Error bars have a width of two standard errors

No statistically significant difference in registered sickness absence was seen between PREVSAM and TAU regarding proportion of individuals who remained in full- or part time work. When all diagnoses were included in the analysis, 67.7% of participants in PREVSAM and 60.7% in TAU remained in work for the entire follow-up period (OR for intervention: 0.735, 95% CI 0.436–1.235, *p* = 0.246). Amongst participants with diagnosis related to “Diseases of the musculoskeletal system and connective tissue” (M), “Injuries, poisonings, and certain other consequences of external causes” (S), “Mental and behavioural disorders” (F) or “Symptoms, signs of disease and abnormal clinical and laboratory findings not elsewhere classified” (R) approximately 75% of the participants remained in work. When only M and S diagnoses were considered, approximately 80% remained in work. See Fig. 2.

The registered sickness benefit gross days and net days are reported in Table 4. The differences between the groups were not statistically significant. However, the PREVSAM group showed a non-significant trend toward fewer net days: approximately 10 days fewer in the full sample, 3.5 days fewer amongst those with M, S, F, R diagnoses, and 1.5 days fewer amongst those with only M and/or S diagnoses during the 12-month follow-up period.

**Table 4:** Comparisons of registered sickness benefit days, gross and net, between the PREVSAM group and TAU, using a zero inflated negative binomial regression model, presenting p-values for the mean amount of registered sickness benefit days for nonzero counts

Time period	PREVSAM group, *n* = 127				TAU, *n* = 122				*p*-value
	mean	sd	median	Q1;Q3	Mean	sd	median	Q1;Q3	
Sickness absence twelve months following baseline, all diagnoses gross days	27.74	64.22	0	0;10.50	36.70	85.95	0	0;20.44	0.839
Sickness absence twelve months following baseline, all diagnoses net days	22.86	55.18	0	0;9	32.41	78.76	0	0;18.46	0.647
Sickness absence twelve months following baseline, M, S, F and R-diagnoses gross days	25.41	62.39	0	0;1.50	27.94	79.26	0	0;2.99	0.752
Sickness absence twelve months following baseline, M, S, F and R diagnoses net days	20.59	52.99	0	0;1.50	24.06	71.75	0	0;2.99	0.608
Sickness absence twelve months following baseline, M and S-diagnoses gross days	23.30	61.72	0	0:0	23.15	75.17	0	0:0	0.869
Sickness absence twelve months following baseline, M and S-diagnoses net days	19.20	52.82	0	0;0	20.80	69.26	0	0;0	0.897

*sd* standard deviation, Q1;Q3 = first and third quartile

PREVSAM “Prevention of sickness absence for musculoskeletal disorders”

TAU “Treatment as usual”

M “Diseases of the musculoskeletal system and connective tissue”

S “Injuries, poisonings, and certain other consequences of external causes”

F “Mental and behavioural disorders”

R “Symptoms, signs of disease and abnormal clinical and laboratory findings not elsewhere classified”

Neither did we see any statistically significant differences between the groups in time to first sickness absence spell. The odds ratios for PREVSAM versus TAU were: all diagnoses OR 0.75 (95% CI 0.49–1.15, *p* = 0.183); M, S, F, R diagnoses OR 0.95 (95% CI 0.58–1.56, *p* = 0.846); and only M and/or S diagnoses OR 0.93 (95% CI 0.53–1.61, *p* = 0.785), although the Kaplan–Meier curves for PREVSAM tended to remain higher over time, indicating a longer time to first sickness absence compared with TAU (Fig. 4a-c in Appendix 1).

#### Self-reported sickness absence.

**Fig. 3:**
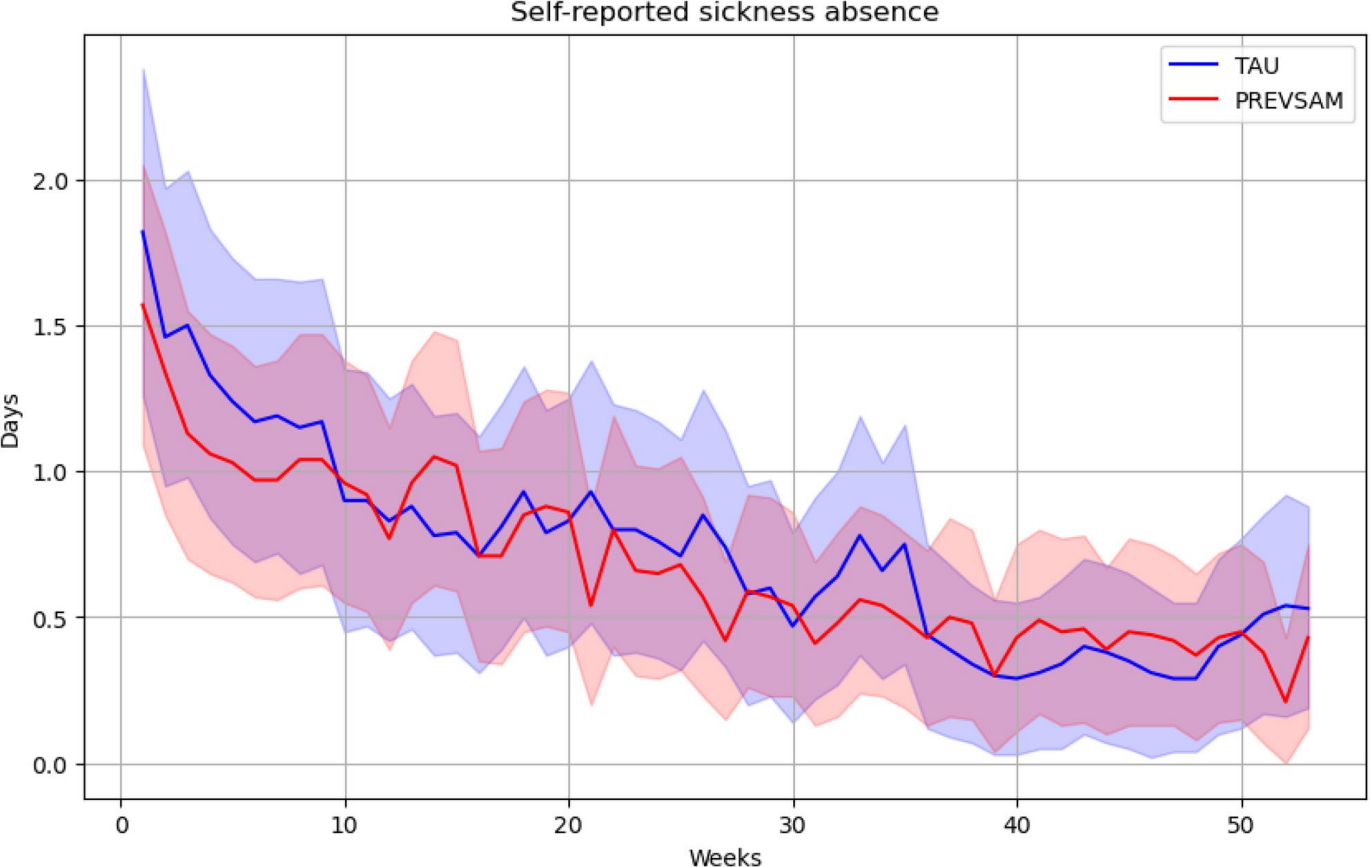
Descriptives of self-reported sickness absence for the PREVSAM and TAU groups reported as days per week (95% CI) by SMS-tracks

Descriptives of self-reported sickness absence for the PREVSAM and TAU groups are shown in Fig. 3. The most common weekly response to the project’s text messages was no days of sickness absence. Most self-reported sickness absence occurred during the first few weeks and decreased over time in a similar pattern in both groups. There were considerable amounts of missing data in both groups, approximately 16% and 21% in the PREVSAM and TAU group respectively, but the level remained fairly constant during the first six months. A slight increase was observed in the following six months, with consistently somewhat more missing data in the TAU group than in the PREVSAM group.

#### Secondary outcomes

No statistically significant differences between the groups were found for any of the secondary outcomes (Table 5). Participants in both the PREVSAM and the TAU group made clinically and statistically significant improvements over time in all PROMs (Table 6 and illustrated in Appendix 2 - Fig. 5a-i). Both groups reported satisfactory self-efficacy and few depressive symptoms already at baseline.

**Table 5:** Differences in groups over time effect in patient-reported health outcome measures (PROMs), repeated measures mixed model analysis with interaction between randomisation and time

Patient-reported health outcomes measure	Fixed effects Generalised Linear Mixed Model with interaction term
Instrument/ dependent variable	Randomisation*Time *p*-value
ÖMPSQ-SF	0.827
WAS	0.957
DRI	0.883
PNRS	0.822
Pain frequency	0.386
PSEQ-2SV	0.289
GSE	0.981
HADS-A	0.318
HADS-D	0.346
EQ VAS	0.547

*ÖMPSQ-SF* Örebro Musculoskeletal Pain Screening Questionnaire Short Form, *WAS* single-item Work Ability Score, *DRI* Disability Rating Index, *PNRS* pain numeric rating scale, *PSEQ-2* pain self-efficacy questionnaire 2-items, *GSE* General Self-Efficacy scale, *HADS* Hospital Anxiety Depression Scale, *EQ VAS* The European Quality of Life Visual Analogue Scale

**Table 6:** Groups over time effect in patient-reported health outcome measures (PROMs), Fixed Effects Mixed Model Analysis without interaction between randomisation and time

Patient-reported health outcomes measure	Fixed effects Mixed Model Analysis *p*-value	
Instrument/dependent variable	Randomisation *p*-value	Time *p*-value
ÖMPSQ-SF	0.948	< 0.001
WAS	0.677	< 0.001
DRI	0.882	< 0.001
PNRS	0.212	< 0.001
Pain frequency	0.627	< 0.001
PSEQ-2SV	0.214	0.001
GSE	0.954	0.028
HADS-A	0.327	0.002
HADS-D	0.224	0.001
EQ VAS	0.851	0.005

*ÖMPSQ-SF * Örebro Musculoskeletal Pain Screening Questionnaire Short Form, *WAS* single-item Work Ability Score, *DRI* Disability Rating Index, *PNRS* pain numeric rating scale, *PSEQ-2* pain self-efficacy questionnaire 2-items, *GSE* General Self-Efficacy scale, *HADS* Hospital Anxiety Depression Scale, *EQ VAS* The European Quality of Life Visual Analogue Scale

## Discussion

This study evaluated effects over a 12-month follow-up period of a team-based intervention for patients with musculoskeletal pain identified as being at increased risk for sickness absence. The hypothesis—that the PREVSAM model would prevent sickness absence to a greater extent, manifested by a higher proportion of participants remaining in full- or part-time work, fewer registered sickness benefit days, and/or a longer time to first sickness absence spell —was not confirmed. Although slightly better outcomes were generally observed in the PREVSAM group, the differences were not statistically significant. As expected, improvements over time in patient-reported health outcomes were seen in both groups, but no significant between-group differences were found.

### Rehabilitation

Early identification of patients with psychological risk factors for developing long-term problems and sickness absence is an essential component of the PREVSAM model. The screening was done using the ÖMPSQ-SF [20]. In previous research, three risk categories based on total score have been used: low risk (up to 40 points), medium risk (41 − 50 points), and high risk (51 points and above) [30]. In line with a previous primary care study [31], we used 40 points as an early-risk threshold to identify patients at risk for developing work disability. However, it is possible that the ÖMPSQ may not optimally capture risk stratification at this early stage, or that a cut-off of 40 is too low for this particular population. Although additional analyses using a higher cut-off (e.g., 50 points) might have provided further clarity, the study was not sufficiently powered to support such subgroup investigations. Another possibility is that only a very small fraction of truly high-risk patients can be identified at this early stage of pain development, limiting the ability of any cut-off to detect between-group differences. However, our qualitative studies revealed that, both according to healthcare professionals and patients who had participated in the PREVSAM intervention, the need for team-based interventions relied on more than the screening results [32, 33]. As many patients benefited from physiotherapy alone, further research is needed to distinguish the group for whom teamwork may be an advantage. Involving multiple healthcare professions may have contributed to a nocebo effect and medicalisation for some participants, by implying that their condition was more serious than initially believed. Although this was not evident in our qualitative studies, the timing of introducing a health plan may have been suboptimal given the individual’s life circumstances and could potentially have influenced the perceived need for team-based interventions [32, 33]. Moreover, as the RCT was mainly conducted during the COVID-19 pandemic, unique factors may have prolonged the start of treatment and affected the possibility of teamwork due to high workload and higher sickness absence amongst both patients and healthcare professionals.

One of the essential components, a health plan, was co-created by the physiotherapist, the occupational therapist, and the patient to a high degree (> 85%) in the PREVSAM group. In the TAU group, an individual rehabilitation plan, co-created by one healthcare professional and the patient, was conducted for only 16% of the participants. A growing body of evidence suggests that a person-centred approach, focusing on a respectful partnership with shared decision-making and goal setting documented in a health plan, is effective for patient satisfaction and health outcomes [34, 35]. However, in the present study, this did not lead to better effects on either sick leave or PROMs compared to TAU.

Goal setting is widely used in rehabilitation, and both benefits and challenges have been reported in previous research. On the one hand, goal setting may improve communication between the patient and the healthcare professionals, clarify what is meaningful to the patient, and increase compliance to treatment strategies [36, 37]. On the other hand, barriers to goal setting have been reported at the patient, healthcare professional, and organisation level [37–39]. From the patients’ perspective, not everyone may want to set goals, or some may perceive that the goals lack personal relevance—and are primarily defined by healthcare professionals [39]. Earlier research has reported time constraints and lack of skills as barriers perceived by healthcare professionals [40], and formulating coping strategies and follow-up actions are frequently missed [37]. On a societal level, the goals may conflict with how healthcare is organised [37–39]. These experiences of goal setting are in line with the findings of our focus group study on PREVSAM healthcare professionals’ views of the model’s benefits and limitations [33]. There are high demands on healthcare professionals in primary care regarding high availability and providing high quality care. About a quarter of the healthcare professionals stated that the health plans were not revised, even when not working satisfactorily [19]. Some PREVSAM team healthcare professionals may have had high expectations for their patients to have sufficient health literacy with capacity to self-manage once the health plan was co-created. However, as many patients at the same time faced other personal challenges in life, some lacked energy to make health-related changes in many areas [32, 33].

The mean number of treatment sessions was higher in the PREVSAM group than in TAU, which, given that PREVSAM involved two professions, is not surprising. The PREVSAM model suggests a time-limited treatment period with structured, individualised interventions and frequent follow-ups; however, person-centred health plans may be very different. Treatment periods from one to 64 weeks were reported, with a median of five sessions with a physiotherapist and two with an occupational therapist, followed by zero to seven follow-ups by phone during the treatment periods. Providing a more comprehensive rehabilitation did not lead to statistically significant differences between the groups in treatment period length. Given that the studied population often had multiple health complaints, the number of treatment sessions was low in both groups. A similar number of treatment sessions has been reported in an earlier study on cognitive functional therapy for chronic low back pain [41].

### Prevention of sickness absence

Both groups reduced self-assessed risk for sickness absence significantly, as measured by the ÖMPSQ-SF. About 60% of both groups scored below the cut-off of 40 points on the ÖMPSQ-SF at the 12-month follow-up. Regarding perceived work ability, the median score increased from 6 to 9 in the PREVSAM group, indicating a transition from poor to good work ability, while the median score in the TAU group increased from 7 to 10, indicating a transition from moderate to excellent work ability according to accepted classifications of the instrument [42]. The WAS instrument as a work ability measure has been suggested to measure other aspects of work ability than sickness absence, as patients may be working despite experiencing reduced work ability [43].

To prevent sickness absence, an essential, albeit optional, component of the PREVSAM model was the collaboration with the workplace or other stakeholders. Studies have shown that a poor work environment poses a risk of ill health and sick leave, however, the risk appears to be higher for people seeking treatment for mental health problems than for those with musculoskeletal diagnoses [44–46] As approximately 30% of the participants in the PREVSAM group had sickness benefit days registered for any period during the 12 months’ follow-up, it can be noted that workplace collaboration was rarely conducted and did not affect the proportion of sick leave in the groups. According to participants’ experiences, integrity prevented this collaboration for some of them, while others already had good collaboration with their employer or did not perceive that their work ability was affected by their health problems [32]. Our focus group study revealed that for many participants, work-related advice on their rights and on the employer’s obligations were considered sufficient [33].

### Study limitations

Conducting a pragmatic RCT in primary care presents several challenges. A pragmatic approach focusses on evaluating whether an intervention is effective under real-life conditions. In this study, the intervention not only involved treatments for MSDs but also included a preventive approach to reduce sickness absence. One key challenge was ensuring the research was relevant and appealing to both participating patients and healthcare professionals, while successfully recruiting enough participants from the target population [47]. Evaluating a complex intervention places a considerable burden on both staff and patients. For the staff, participation in the study required the implementation of new routines and workflows to deliver the intervention and to collect relevant evaluation data. For patients, it added the additional task of completing extensive questionnaires. Another challenge was recruiting enough participants to ensure adequate statistical power. Although no group differences were observed at the 5% significance level, the risk of a Type II error is considerable, as the study was disrupted by the COVID-19 pandemic and did not reach its planned sample size. Pragmatic trials of complex interventions require larger samples because flexibility in delivery and uptake increases variability, while our primary outcome, sick leave, is also difficult to measure reliably due to its variability and many non-events. Nevertheless, the pragmatic design enhances external validity and ensures that the findings are relevant to routine clinical practice.

The process evaluation of the PREVSAM trial indicated that the PREVSAM model’s essential components were implemented for most participants [19]. However, both physiotherapy and occupational therapy assessments before a team meeting to create a joint health plan should have been provided to all patients. This was not the case, which may have resulted in an insufficient distinction between the intervention and the control condition, limiting our ability to detect differences in effect between the groups.

Treatment fidelity may have been influenced by several factors. The intervention required active engagement from both healthcare professionals and patients. Organisational constraints, such as high workload, time pressure and a perceived lack of support from colleagues and managers, may have affected the conditions for a team-based, person-centred, approach. Moreover, the COVID-19 pandemic, with strict stay-at-home recommendations, increased absenteeism amongst both patients and staff. Scheduled consultations were often cancelled at short notice and could not always be rescheduled. According to previous research, low compliance can render an effective intervention ineffective [48]. Whether this may have influenced the outcomes of the PREVSAM intervention should be considered.

### Clinical implications and future research

The participants constituted a heterogeneous group, both in terms of the type of MSDs, the nature of their work, and other characteristics, and most remained in work. They experienced improvements in patient-reported outcomes over time, highlighting the recovery potential of many individuals with musculoskeletal disorders. Standard care, when accessible and appropriately delivered, may be sufficient for a large proportion even within this population considered to be at elevated initial risk.

In literature, workplace interventions including modifications to the physical environment or equipment, alterations in work design or organisation, and adjustments to working conditions have demonstrated various effects on work ability. These interventions have, in some studies, been associated with reductions in sickness absence among workers with MSDs when compared with TAU [49]. In this study, when the MSD affected work ability, discussions concerning rights and obligations at the workplace were perceived as helpful, even though many did not want their employer to be aware of their condition, although some later regretted it [50]. Most participants felt confident addressing work‑related problems directly with their employer after this guidance, and only a few wished for the team to contact the employer; none requested a tripartite meeting. In standard occupational and physiotherapy practices, discussions about rights and obligations at the workplace and contacting employers for dialogue or conducting workplace visits, is not included. To provide this work-related information to those rather few in need may still be a valuable task to prevent sickness absence in MSD care.

From a clinical perspective, and in alignment with biopsychosocial and person-centred approaches, we propose that rehabilitation for patients with MSDs should incorporate flexible Person-Centred Coordinated Care Pathways. These should offer physiotherapy alone or interdisciplinary team-based rehabilitation, depending on patients’ needs and preferences, with a focus on identifying which subgroups truly benefit from additional or tailored interventions. More research is warranted to explore whether systematic screening can support more efficient, person-centred rehabilitation. Is it possible to refine prognostic tools to more accurately distinguish between individuals likely to recover and those at genuine risk of chronicity and long-term work absence—while also assessing their resources, motivation, and capacity to engage in rehabilitation? Could this patient group be characterised by particular distinguishing features? Further studies using longitudinal or prospective designs are needed to evaluate sickness absence, health-related outcomes, and cost-effectiveness for at-risk patients with MSDs.

## Conclusions

The PREVSAM model was not shown to be more effective than TAU in reducing sickness absence amongst patients with musculoskeletal disorders who were identified to be at risk for long-term sick leave. The findings from the study are nevertheless encouraging as most participants—regardless of group—remained in full- or part-time work and had no registered sickness benefit days during the 12 months’ follow-up. This finding suggests that most individuals who, in primary care, initially present with risk factors for long-term pain and sick leave do not go on to develop prolonged sickness absence.

Furthermore, patient-related health outcomes improved significantly over time in both groups, suggesting that the interventions, regardless of type, contributed positively to participants’ health and recovery. These results highlight the resilience of a substantial proportion of this patient population and underscore the importance of further development of risk assessment and tailored support.

## Supplementary Information

Supplementary Material 1.

Supplementary Material 2.

## Data Availability

The datasets used and analysed in the current study are available from the corresponding author on reasonable request.

## Abbreviations

RCT: Randomised controlled trial
PREVSAM: PREVention of sickness absence for musculoskeletal disorders
TAU: Treatment as usual
MSD: Musculoskeletal disorders
ÖMPSQ: SF–the Örebro musculoskeletal pain screening questionnaire, short form
SSIA: Swedish social insurance agency
PROM: Patient–reported health outcome measure
