# Supplementary material for: Prevention of sickness absence through early identification and rehabilitation of at-risk patients with musculoskeletal disorders (PREVSAM): 12-month follow-up of a randomised controlled trial
Source: BMC Musculoskelet Disord. 2026 Apr 27;27:360. doi: 10.1186/s12891-026-09859-x (PMC13123168; doi:10.1186/s12891-026-09859-x)
Supplement: Supplementary file 1 — Supplementary Material 1. [file 12891_2026_9859_MOESM1_ESM.docx]

Figures 4a-4c

Cox regression Kaplan-Meier curves


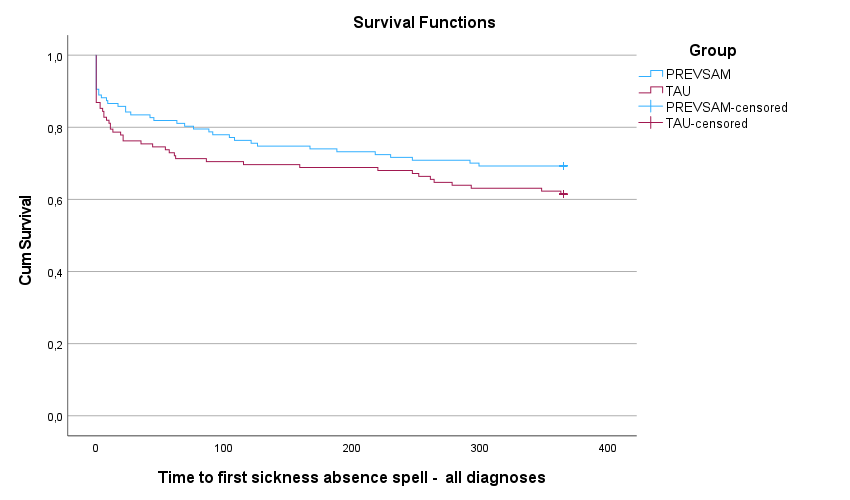


Figure 4a - Time to first sickness absence spell for all diagnoses carried out with Cox regression. Kaplan-Meier curves for PREVSAM and TAU groups are given. PREVSAM lies higher towards the left of the x-axis as a larger amount of PREVSAM subjects had longer times until first sick absence. No significant difference between groups was found (OR for PREVSAM 0.75, 95% CI 0.49 - 1.15, p=0.183).


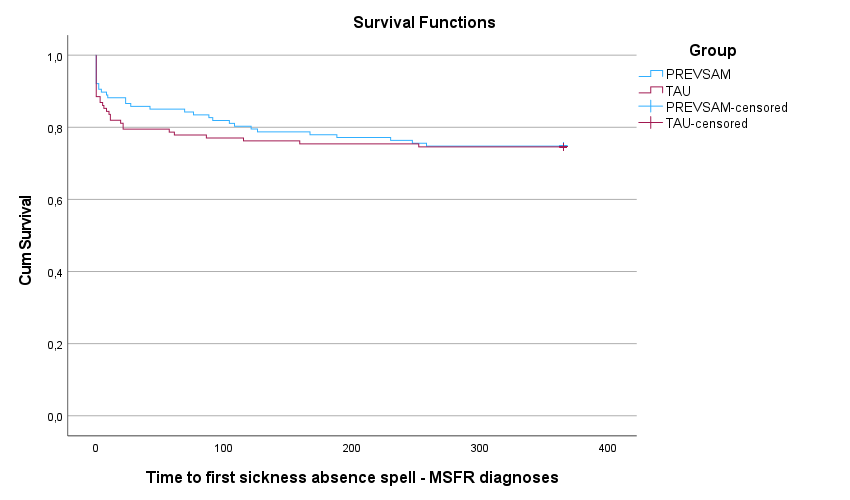


M “Diseases of the musculoskeletal system and connective tissue”

S “Injuries, poisonings, and certain other consequences of external causes”

F “Mental and behavioural disorders”

R “Symptoms, signs of disease and abnormal clinical and laboratory findings not elsewhere classified”

Figure 4b - Time to first sickness absence spell for all diagnoses carried out with Cox regression. Kaplan-Meier curves for PREVSAM and TAU groups are given. PREVSAM lies higher towards the left of the x-axis as a larger amount of PREVSAM subjects had longer times until first sick absence. No significant difference between groups was found (OR for PREVSAM 0.95, 95% CI 0.58 - 1.56, p=0.846).


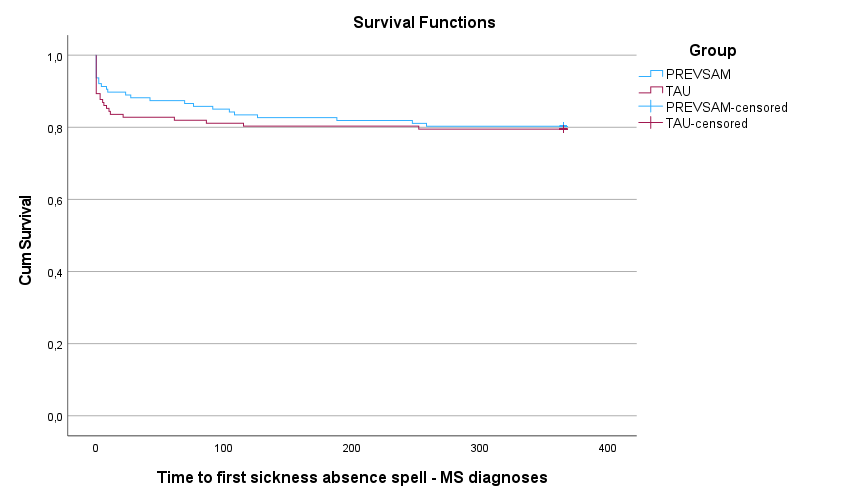


M “Diseases of the musculoskeletal system and connective tissue”

S “Injuries, poisonings, and certain other consequences of external causes”

Figure 4c - Time to first sick absence spell for all diagnoses carried out with Cox regression. Kaplan-Meier curves for PREVSAM and TAU groups are given. PREVSAM lies higher towards the left of the x-axis as a larger amount of PREVSAM subjects had longer times until first sick absence. No significant difference between groups was found (OR for PREVSAM 0.93, 95% CI 0.53 - 1.61, p=0.785).
