# Supplementary material for: Prevention of sickness absence through early identification and rehabilitation of at-risk patients with musculoskeletal disorders (PREVSAM): 12-month follow-up of a randomised controlled trial
Source: BMC Musculoskelet Disord. 2026 Apr 27;27:360. doi: 10.1186/s12891-026-09859-x (PMC13123168; doi:10.1186/s12891-026-09859-x)
Supplement: Supplementary file 2 — Supplementary Material 2. [file 12891_2026_9859_MOESM2_ESM.docx]

Appendix 2 Groups over time effect for patient-reported outcome measures (PROMs)


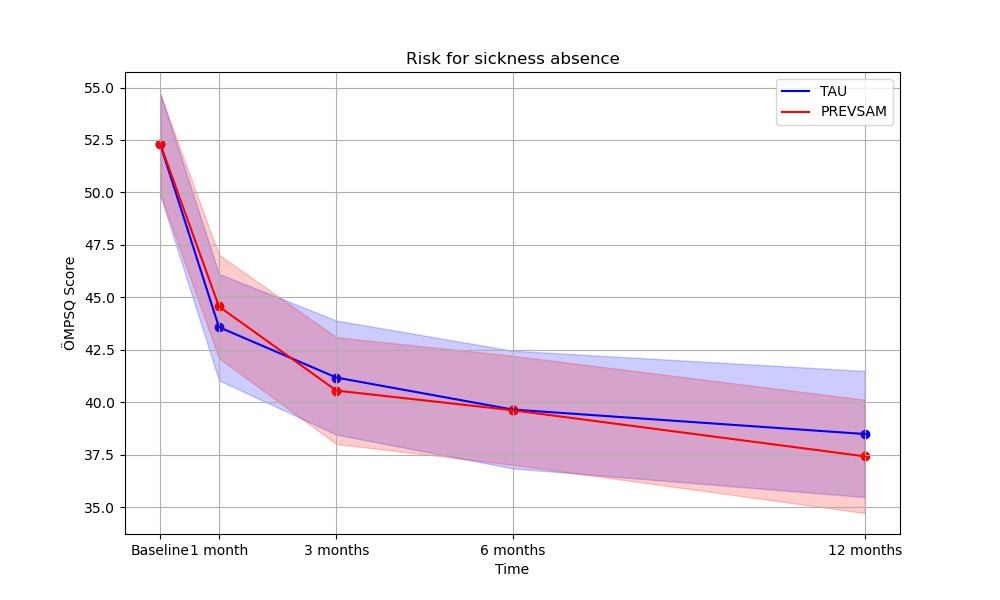


Figure 5a. Risk for sickness absence measured with Örebro Musculoskeletal Pain Screening Questionnaire Short Form


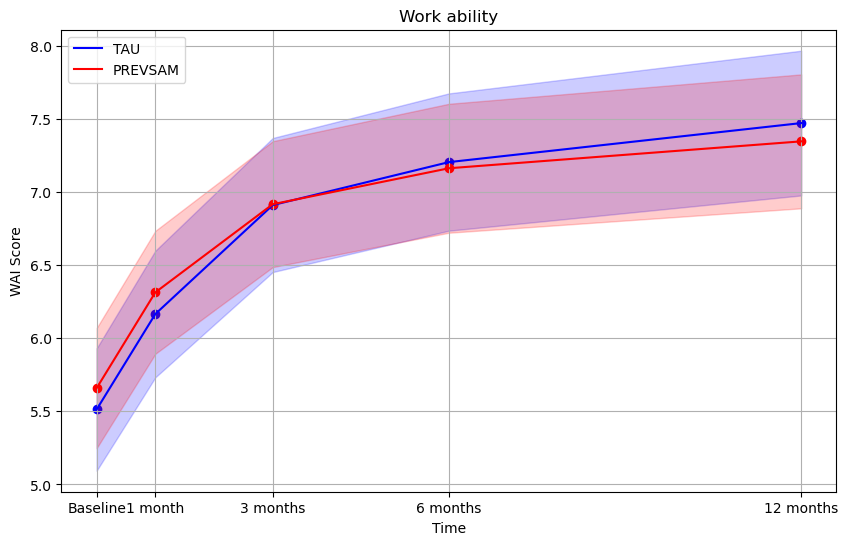


Figure 5b. single-item Work ability Score


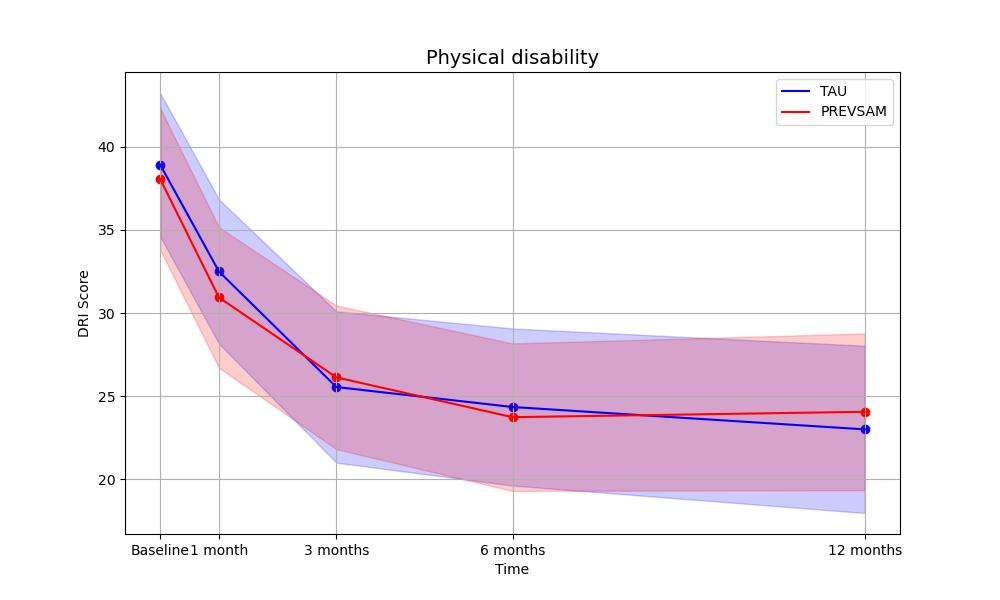


Figure 5c. Physical disability measured by Disability Rating Index


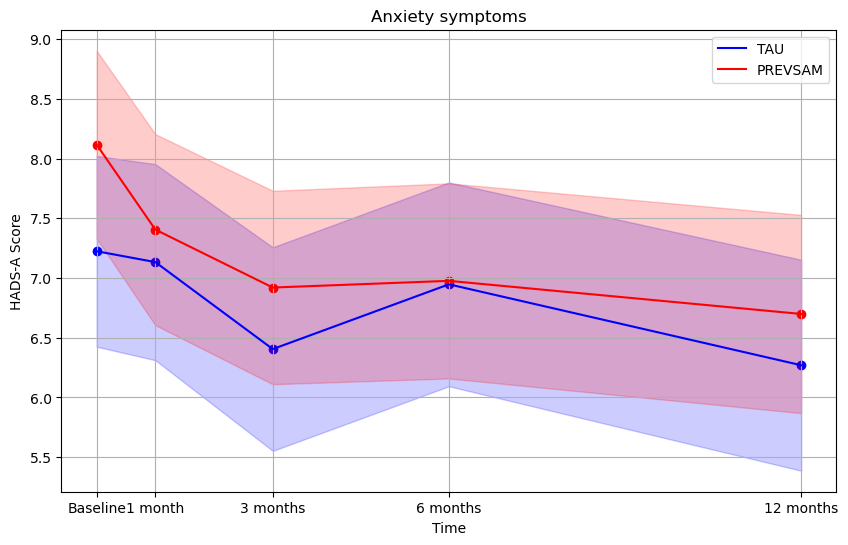


Figure 5d. Anxiety symptoms measured by Hospital Anxiety Depression Scale - Anxiety


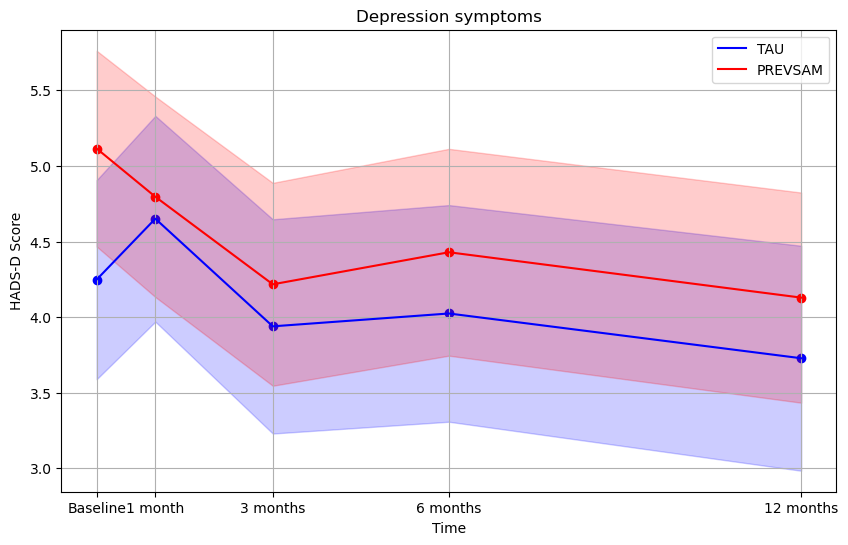


Figure 5e. Depression symptoms measured by Hospital Anxiety Depression Scale - Depression


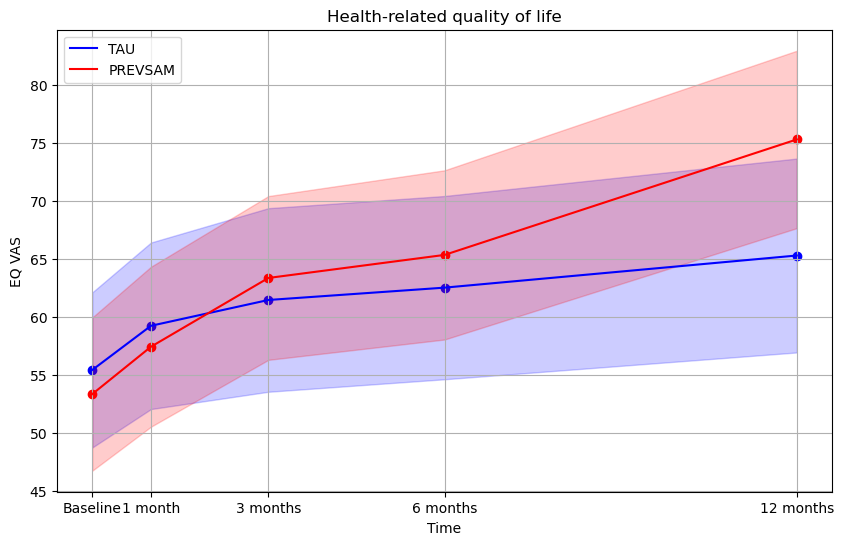


Figure 5f. Health-related quality of life measured by The European Quality of Life Visual Analogue Scale


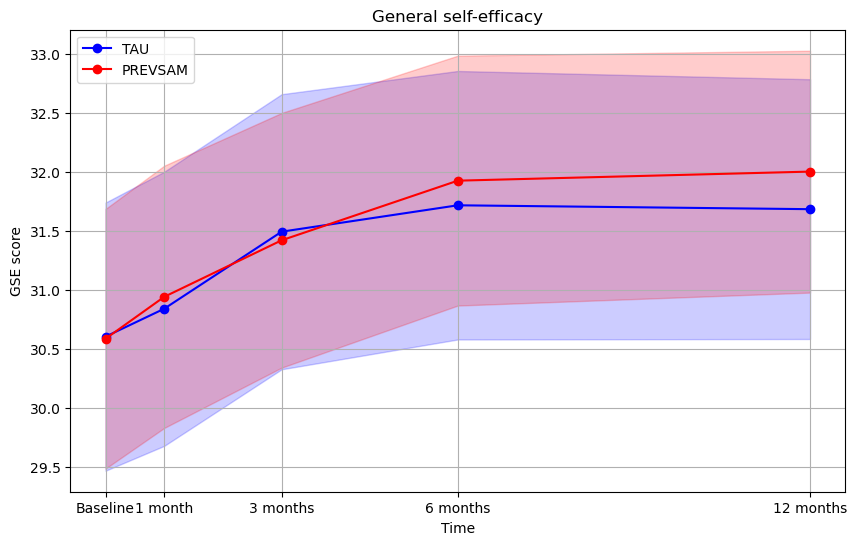


Figure 5g. General self-efficacy Scale


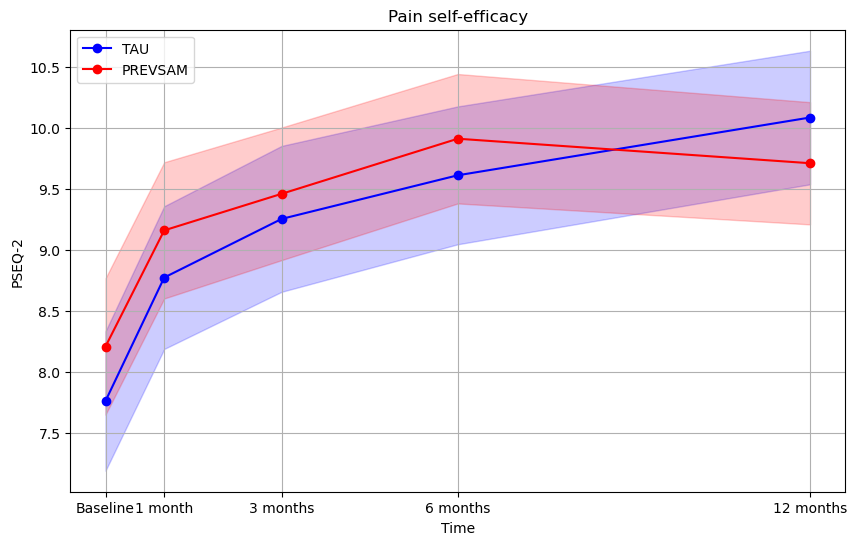


Figure 5h. Pain self-efficacy measured by Pain self-efficacy questionnaire 2-items


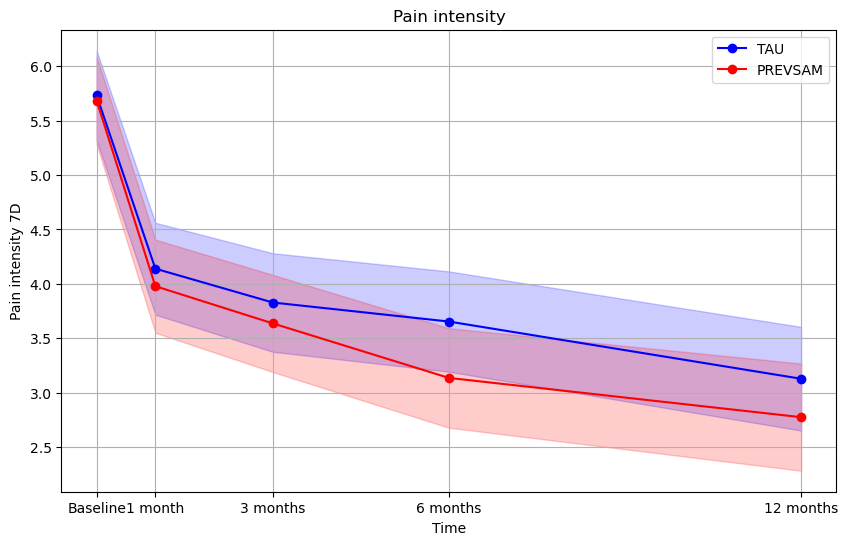


Figure 5i. Pain intensity over 7 days, pain numeric rating scale

Figure 5a-i. Changes from baseline to 12 months in patient-reported outcomes in the PREVSAM and TAU groups. Coloured bands represent 95% confidence intervals analysed using generalised linear mixed models (p=ns)
